# Supplementary material for: Enhancing the Anti-Migration Performance and Mechanical Properties of EPDM Insulation through Functionalized GO
Source: Polymers (Basel). 2023 Mar 30;15(7):1731. doi: 10.3390/polym15071731 (PMC10097289; doi:10.3390/polym15071731)
Supplement: Supplementary file 1 [file polymers-15-01731-s001.zip › polymers-2276428-supplementary.pdf]

Supporting Information

Enhancing the Anti-Migration Performance and Mechanical Properties of EPDM Insulation through Functionalized GO

Zhehong Lu [1], Ziqiang Zhu [1], Yulong Zhang [2], Chenyang Wang [1], Haoran Bai [3], Guangpu Zhang [1], Yubing Hu [1,*] and Wei Jiang [1,*]

[1] National Special Superfine Powder Engineering Research Center of China, Nanjing University of Science and Technology, Nanjing 210014, China
[2] China North Industry Advanced Technology Generalization Institute, Beijing100089, China
[3] College of Materials Science and Engineering, Shenyang University of Technology, Shenyang 110870, China
* Correspondence: hyb@njust.edu.cn (Y.H.); superfine_jw@126.com (W.J.)

Experimental

2.1. Materials

Natural graphite powder with an average particle size of 400 mesh and purity of >99% was supplied by Qingdao Tianhe Graphite Co., Ltd., China. Potassium permanganate ($KMnO_4$, AR), concentrated sulfuric acid (H_2SO_4, 95–98%), triethylamine, ethanol, and tetrahydrofuran (THF), all analytical-grade, hydrochloric acid (37%), and sodium nitrate were all supplied by Sinopharm Chemical Reagent Co., Ltd, China. Ethylene propylene diene monomer (Keltan8550, 55 wt.% ethylene and 5.5 wt.% 5-ethylidene-2-norbornene (ENB)) was purchased from Lanxess Co., Ltd., Germany. Triallylisocyanurate (TAIC), zinc oxide, and stearic acid were supplied by Chengdu Chron Chemical Co., Ltd., $NH_3 \cdot H_2O$, 3-aminopropyltriethoxysilane, octadecylamine (ODA), dicumyl peroxide (DCP), and antioxidant 1010 were provided by Aladdin Reagent Shanghai Co., Ltd., China.

2.2. Synthesis of AGO and HGO

GO (1.00 g) and 1.00 mL KH550 were dispersed in methylbenzene in a 200-mL four-neck flask. The reaction solution was heated at 80 °C to reflux for 7 h under mechanical stirring, and then it was filtered through a 0.45-μm filter. The obtained material was washed in order by distilled water, methylbenzene and acetone. Finally, the material was dried at 60 °C in vacuum drying oven for 24 h, and it was named AGO.

400 mg GO was dispersed in deionized water by ultrasound and 600 mg ODA was ultrasonically dispersed in ethanol. Then mix the solutions together and reacted at 90°C for 15 hours. The product was washed by water and ethanol several times, and finally dried in a freeze dryer, and it was named HGO.

Immersion absorption method

The migration experiment was carried out by the immersion absorption method, which refers to immersing the test material in the component liquid and regularly taking out the weighing mass until the material weight does not increase or the sample is destroyed. It is a simple and accurate method which can quickly observe the migration of components and intuitively compare the anti-migration effects of adding different fillers.

In this work, we will conduct migration tests with DOP at 25°C, 40°C, 60°C, and 80°C. The size of EPDM sheet is prepared as 30×30×2 mm3. The migration concentration at time t can be calculated according to the following equation:

$$\mathrm{A} = \frac{m_t - m_0}{m_0} \times 100\% \tag{1}$$

where A represents the migration concentration of DOP, m_t represents the mass of the diffused substance sheet including the absorbed substance, m_0 represents the initial mass of sheet.
